# BNIP3 Downregulation Ameliorates Muscle Atrophy in Cancer Cachexia

**DOI:** 10.3390/cancers16244133

**Published:** 2024-12-11

**Authors:** Claudia Fornelli, Marc Beltrà, Antonio Zorzano, Paola Costelli, David Sebastian, Fabio Penna

**Affiliations:** 1 Department of Clinical and Biological Sciences, University of Torino, 10043 Turin, Italy; claudia.fornelli@unito.it (C.F.); paola.costelli@unito.it (P.C.); 2 Institute for Research in Biomedicine (IRB Barcelona), The Barcelona Institute of Science and Technology, 08028 Barcelona, Spain; marc.beltra@irbbarcelona.org (M.B.); antonio.zorzano@irbbarcelona.org (A.Z.); 3 Department of Biochemistry and Physiology, School of Pharmacy and Food Sciences, University of Barcelona, 08028 Barcelona, Spain; dsebastian@ub.edu; 4 Institute of Biomedicine of the University of Barcelona (IBUB), University of Barcelona, 08028 Barcelona, Spain; 5 Centro de Investigación Biomédica en Red de Diabetes y Enfermedades Metabólicas Asociadas (CIBERDEM), Instituto de Salud Carlos III, 28029 Madrid, Spain

**Keywords:** cancer cachexia, mitochondria, mitophagy, BNIP3, muscle wasting

## Abstract

Background and Aims: Cancer cachexia is a complex syndrome affecting most cancer patients and is directly responsible for about 20% of cancer-related deaths. Previous studies showed muscle proteolysis hyper-activation and mitophagy induction in tumor-bearing animals. While basal mitophagy is required for maintaining muscle mass and quality, excessive mitophagy promotes uncontrolled protein degradation, muscle loss and impaired function. BNIP3, a key mitophagy-related protein, is significantly increased in the muscles of both mice and human cancer hosts. This study aimed to define the potential of mitigating mitophagy via BNIP3 downregulation in preserving mitochondrial integrity, counteracting skeletal muscle loss in experimental cancer cachexia. Methods: Two in vivo gene delivery methods were performed to knock down muscle BNIP3: electroporation of a BNIP3-specific shRNA expression vector or adenovirus injection. Results: The electroporation effectively reduced muscle BNIP3 in healthy mice but was ineffective in C26 tumor-bearing mice. In contrast, adenovirus-mediated BNIP3 knockdown successfully decreased BNIP3 levels also in tumor hosts. Although BNIP3 knockdown did not impact overall on body or muscle mass, it improved muscle fiber size in C26-bearing miceh2, suggesting partial prevention of muscle atrophy. Mitochondrial respiratory chain complexes (OxPhos) and TOM20 protein levels were consistently rescued, indicating improvements in mitochondrial mass, while H_2_O_2_ levels were unchanged among the groups, suggesting that BNIP3 downregulation does not impair the endogenous control of oxidative balance. Conclusions: These findings suggest that a fine balance between mitochondrial disposal and biogenesis is fundamental for preserving muscle homeostasis and highlight a potential role for BNIP3 modulation against cancer-induced muscle wasting.

## Simple Summary

Cancer patients frequently develop a syndrome named cachexia that causes severe muscle loss and frailty, eventually representing the cause of death. Muscle atrophy and muscle weakness are characterized by massive degradation of endogenous proteins, potentially consequent to excessive disposal of mitochondria through the selective autophagic process of mitophagy. This study explored whether selectively silencing BNIP3, a mitophagy-related protein upregulated in the muscle of both mouse and human cancer hosts, could help in preventing muscle loss. Two distinct methodological silencing approaches were tested, either by electroporation of a plasmid or via adenoviral particle injection. Although the first method was ineffective in tumor-bearing mice, the adenovirus-based approach significantly reduced BNIP3 levels and moderately increased muscle fiber size, suggesting partial prevention of muscle atrophy. BNIP3 silencing also maintained mitochondrial mass without disrupting oxidative balance, highlighting BNIP3’s central role in cancer cachexia and suggesting that targeting BNIP3 may help in supporting muscle health in cancer patients.

## 1. Introduction

Cancer cachexia is a complex multifactorial syndrome that affects more than half of all cancer patients, being directly responsible for about 30% of cancer deaths [1,2,3]. Involuntary loss of body weight, associated with massive adipose tissue and muscle wasting together with systemic inflammation and energy metabolism alterations represent typical hallmarks of this syndrome [1,2,3]. Moreover, cancer cachexia is often accompanied by anorexia, asthenia and fatigue, leading to reduced tolerance for anticancer treatment and increased infection susceptibility, impacting health care costs and patients’ quality of life [1,2,3]. Previous studies in tumor-bearing animals demonstrated the key role played by muscle proteolysis activation together with protein biosynthesis decrease in skeletal muscle depletion, highlighting that a fine balance between protein degradation and synthesis is fundamental for preserving muscle homeostasis (reviewed in [1,4]). Two main proteolytic systems are involved in muscle wasting: the ubiquitin–proteasome system (UPS) and the macro autophagy–lysosomal pathway (hereinafter referred to as autophagy) [5,6].

Autophagy is a constitutive self-degradation process of cellular components that, working at basal levels, regulates homeostasis in both physiological and pathophysiological contexts, maintaining protein and organelle integrity and balancing cellular energy and nutrient availability [7,8,9]. It acts by sequestering and encapsulating cytoplasmic constituents within an isolated double-membrane autophagosome and directing them to lysosomal degradation [10]. Autophagy regulation is complex, highly conserved from yeast to humans and tightly modulated depending on the context. Overactivation may occur in response to extra- or intracellular stimuli such as starvation, growth factor deprivation, mitochondrial damage and infections [7,8,9,11]. The relevance of autophagy as a homeostatic mechanism is underscored by the implication of autophagic system defects in a wide range of diseases, including neurodegenerative and metabolic ones, aging and ultimately cancer, where autophagy’s role is highly context-specific according to cell type, tumor stage and microenvironment [12,13,14]. Regarding muscle homeostasis, on one side, a strict modulation of the autophagic system is required for maintaining muscle mass and quality, protecting cells by counteracting oxidative stress and removing damaged proteins and mitochondria, while on the other side, excessive or defective autophagy can promote cellular degradation, worsening muscle loss and finally impairing muscle function [11,14,15].

Mitophagy, observed in mammalian cells for the first time by electron microscopy, is a selective form of autophagy responsible for removing old or damaged mitochondria in order to preserve the cellular homeostasis through the maintenance of mitochondrial pool integrity [10,16,17,18]. Dysfunctional mitochondria show a reduced oxidative phosphorylation capacity and ROS accumulation, resulting in a significant increase in cellular oxidative stress [15,16,17,18]. Furthermore, in the absence of an efficient clearance by antioxidant systems, ROS accumulation has been shown to directly contribute to skeletal muscle wasting through the activation of various catabolic signaling pathways, including NF-kB and the ubiquitin–proteasome system [19].

A multitude of receptors control mitophagy by sensing mitochondrial depolarization, hypoxia and extracellular signals [20,21,22]. Recent studies reveal a key role in the selective autophagic recognition of damaged mitochondria played by the Pink1–Parkin signaling pathway and the mitophagic receptors Nix (also known as BNIP3L) and BNIP3 [22,23,24]. Among these, BNIP3, an outer mitochondrial membrane protein and a member of the Bcl-2 family of cell death-regulating factors, primarily regulates mitochondrial fragmentation and mitophagy via interaction with the microtubule-associated protein 1A/1B-light chain 3 (LC3), an autophagosome integral protein localized on both the inner and outer autophagosome membrane [20,21,22,24,25,26]. BNIP3 is strongly expressed in the liver, heart and skeletal muscle, where in response to hypoxia or starvation it is highly induced by the binding respectively of the hypoxia-inducible factor-1 (HIF-1) or Foxo3 transcription factor to a site on the BNIP3 promoter [22,24,26,27]. In particular, under those conditions, BNIP3 localizes to mitochondria by its transmembrane domain and exposes its N-terminal domain to the cytosol, anchoring the C-terminal domain to the outer mitochondrial membrane and forming a stable homodimer [24,26,27]. When BNIP3 is induced and forms a homodimer, it triggers the opening of the permeability transition pore (PTP) determining the loss of mitochondrial membrane potential, accompanied by an increase of ROS production and finally leading to cell death [28].

Recent studies showed a significant BNIP3 mRNA increase in experimental models of cancer-induced muscle wasting, reflecting mitophagy induction [29]. BNIP3 upregulation is often accompanied in rodents by increased ROS production and dysfunctional mitochondria, resulting in reduced mitochondrial abundance in the skeletal muscle [29,30]. The present work aimed to exploit the potential of BNIP3 silencing in the skeletal muscle of C26 hosts in order to mitigate mitophagy and preserve mitochondrial pool integrity, potentially improving skeletal muscle mass and function.

## 2. Materials and Methods

### 2.1. Animal Model and Experimental Design

Experimental animals were cared for in compliance with the Italian Ministry of Health Guidelines and the Policy on Human Care and Use of Laboratory Animals (NIH, 1996). The first in vivo experiment was approved by the Bioethical Committee of the University of Torino, Italy, and by the Italian Ministry of Health (authentication number 579/2018-PR, date of approval 31 July 2018) and the second experiment by the Institutional Animal Care and Use Committee of the Barcelona Science Park and University of Barcelona (code CEEA-OH-22-023, date of approval 1 June 2022). The animals were maintained on a regular dark–light cycle, with free access to food and tap water. Two different protocols of gene delivery were performed. In the first experiment, 8-month-old female BALB/c mice were randomized and divided into two groups: healthy controls (*n* = 6) and C26 (*n* = 11). Next, 30 µg (1 µg/µL) of an expression plasmid containing either the BNIP3 inhibitory siRNA-GFP (shBNIP3) sequence or a scramble-GFP (SCR) sequence was injected into the left and right TA muscles, respectively. Transfection was performed by electroporation into skeletal muscle fibers (see below). After two days, mice were anesthetized with isoflurane and subcutaneously inoculated on the back either with 5 × 10^5^ C26 (colon26) adenocarcinoma cells obtained from Prof M.P. Colombo (National Cancer Institute, Milano, Italy) in 2005 (C26 group) or 0.9% saline solution (control group). The animals were sacrificed 14 days after C26 inoculation. In the second experiment, 6-week-old male BALB/c mice were subcutaneously inoculated with 5 × 10^5^ C26 (*n* = 10) or saline solution (*n* = 8), and two days later, a purified mouse BNIP3 shRNA adenovirus was injected into the TA and GSN muscles of one of the hindlimbs. The contralateral hindlimb was injected with purified Ad-null adenovirus, serving as a control. The mice were sacrificed between 12 and 14 days from tumor inoculation according to body weight loss. The muscles were rapidly collected, weighed, frozen in liquid nitrogen and stored at −80 °C for histological and biochemical analyses. In both experiments, mice were monitored daily for signs of distress, and body weight and food intake were recorded every two days.

### 2.2. Plasmid Generation and Isolation

The sequence of a validated shRNA targeting BNIP3 [31] was cloned and inserted into a pcDNA 6.2-GW/EmGFP-miR expression vector (Invitrogen, Waltham, MA, USA, K493600), according to the manufacturer’s instructions. A scramble plasmid, which codes a siRNA sequence that does not target any known vertebrate gene, was used as control. Subsequently, plasmid DNA for electroporation was purified using the QIAGEN Plasmid Maxi Kit (QIAGEN, Hilden, Germany), following the manufacturer’s instructions.

### 2.3. Gene Delivery

#### 2.3.1. Electroporation

Muscle electroporation was performed as in previous experiments with some modifications [14]. In order to obtain a successful gene delivery, 2 h pretreatment with hyaluronidase (0.4 U/µL diluted in sterile saline solution 0.9% NaCl in a total volume of 30 µL/injection) was performed, resulting in an increase of transfection efficiency without affecting whole muscle function, as previously reported [32]. The mice were then anesthetized, the hindlimbs were shaved between the knee and ankle with a razor and a short (less than 1 cm) incision was performed along the tibia. The TA was exposed after fascia removal, and 30 µg of plasmids (1 µg/µL), diluted in sterile half saline solution (0.45% NaCl), was injected slowly into the TA using a 30 G syringe needle (left TA: shBNIP3-GFP plasmid; right TA: SCR-GFP plasmid). Subsequently, one electrode was placed behind the TA muscle and one on top, before delivering 5 electric pulses (50 volts/cm, 200 ms intervals) using an electroporator (ECM-830, BTX-Harvard Apparatus, Holliston, MA)). This procedure was repeated for both hindlimbs. The incision was sutured, and the animals were placed in their cage to recover, where they were monitored every day. No signs of necrosis or inflammation were observed as a result of the procedure. Proper gene delivery was confirmed by evaluating GFP fluorescence, allowing the identification of successfully transfected muscle fibers under a microscope. 

#### 2.3.2. Adenovirus

Adenoviruses were generated in the Viral Vector Production Unit of the Autonomous University of Barcelona. Adenoviral transduction was performed two days after C26 inoculation by intramuscular injection of purified shBNIP3 (Ad-U6-mBNIP3-shRNA, Vector Biolabs, Malvern, PA, USA) adenoviruses. GSN muscles were infected with 2 × 10^9^ infection units (IU)/mL in a volume of 75 μL saline in three different areas to assure the maximal transduction efficiency. TA muscles were infected with a single injection of 6.66 × 10^8^ IU in a volume of 25 µL saline. The contralateral hindlimb muscles were injected with purified Ad-null adenoviruses and served as a control. Muscles were collected 12–14 days after C26 inoculation.

### 2.4. Histology

#### 2.4.1. Hematoxylin and Eosin Staining and Cross-Sectional Area (CSA)

Frozen sections of TA, 10 µm thick, were stained with hematoxylin and eosin following standard procedures, mounted with Eukitt Quick mounting media (Sigma, Macquarie Park, Australia), photographed at 10× magnification and digitally reconstructed by using the GIMP software 2.10.30. Fiber CSA was determined using randomly chosen GFP-positive fibers, 100 myofibers per muscle, using the Image J 1.53k software and expressed in pixels/µm. 

#### 2.4.2. Succinate Dehydrogenase Staining

Frozen sections of TA, 10 µm thick, were incubated for 10 min at 37 °C with a solution containing 1 mg/mL NTB (nitro tetrazolium blue chloride) and 27 mg/mL Na-succinate in phosphate-buffered saline. The whole TA section was photographed at 10× magnification and digitally reconstructed by using the GIMP software 2.10.30. 

### 2.5. Transmission Electron Microscopy (TEM)

Samples were prepared at the Electron Cryomicroscopy Unit of the CCiTUB (University of Barcelona). Briefly, GSN muscles were cut into pieces of ~1 mm^3^ and transferred to glass vials filled with 2% paraformaldehyde and 2.5% glutaraldehyde in phosphate buffer (0.1 M, pH 7) for 24 h at 4 °C. The samples were then washed, post-fixed with 1% osmium tetroxide (EMS, USA) and 0.8% potassium ferricyanide at 4 °C and washed again. Finally, the samples were dehydrated in acetone, infiltrated and embedded with Epon resin (EMS, USA) for 2 days and polymerized at 60 °C for 48 h. Semi-thin sections were observed under a Leica DM2000 light microscope (Leica microsystems, Vienna, Austria) to confirm the longitudinal orientation. Ultrathin sections were obtained using a Leica Ultracut UC6 ultramicrotome (Leica Microsystems, Vienna, Austria) and mounted on Formvar-coated copper grids. They were stained with UA-Zero (Agar Scientific, Stansted, UK) and 3% lead citrate and then observed under a JEOL-1010 electron microscope (Jeol, Akishima, Japan) equipped with an SIS Megaview III CCD camera and the AnalySIS software (version 3.2). At least 20 different TEM images/sections from three mice per experimental group were obtained and quantified.

### 2.6. Protein Expression Analyses

#### 2.6.1. Protein Extraction

Total protein extracts were obtained by homogenization of frozen slices or tissue (10% *w*/*v*) from TA and GSN muscles, respectively, in ice-cold RIPA buffer solution with freshly added protease and phosphatase inhibitors (Sigma cocktails). The samples were sonicated, and the supernatant was collected after centrifugation at 3000 rcf for 5 min at 4 °C. Protein concentration was determined using the Bradford assay (BioRad, Hercules, CA, USA) with bovine serum albumin as a standard. 

#### 2.6.2. Western Blotting

The samples were resolved in Midi PROTEAN TGX Precast Gels (Bio-Rad, Hercules, CA, USA) and then transferred using the Trans-Blot Turbo Transfer System (Bio-Rad, Hercules, CA, USA). The following antibodies were used: BNIP3 (1:1000 Cell Signaling #3769), LC3 (1:1000 Sigma #L7543), GFP (1:800 Santa Cruz #sc9996), OXPHOS (1:1000 Abcam #ab110413) and TOM20 (1:1000 Abcam #ab186735). Vinculin (1:2000 Santa Cruz #sc73614) expression was considered as a loading reference protein. Proteins were detected using the Bio-Rad ChemiDoc and quantified by densitometry with the ImageLab 6.0.1 software.

#### 2.6.3. Amplex™ Red Hydrogen Peroxide/Peroxidase Assay 

All stock solutions were prepared according to manufacturer instructions (Invitrogen, Catalog no. A22188), and samples were serially diluted to determine the optimal amount for the assay. Briefly, 10 µL of total GSN protein extract was added to wells of a 96-well plate, bringing samples to a final volume of 50 µL with the 1× Reaction Buffer working solution; 50 µL of the Amplex^®^ Red reagent/HRP working solution was added to each well containing the standards, controls and samples. After 30 min of incubation at room temperature while protected from light, fluorescence was measured (Ex/Em 530–560/590 nm) at different time points (10, 20 and 30 min). Background fluorescence was corrected by subtracting the value derived from the no-H_2_O_2_ control, and H_2_O_2_ levels were finally obtained, normalizing for protein concentration.

## 3. Results

### 3.1. BNIP3 Is Increased in the Skeletal Muscle of Tumor-Bearing Mice

**Figure 1:**
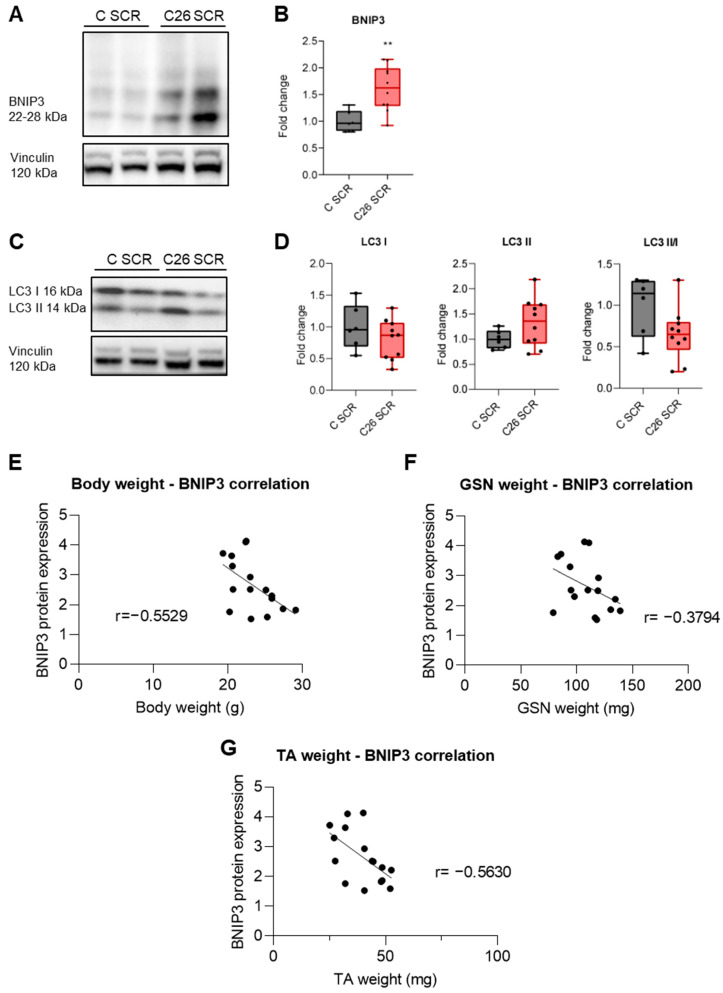
Mitophagy induction in C26 tumor-bearing mice. Representative immunoblotting showing the expression of BNIP3 and LC3-I/II in the skeletal muscle (**A**,**C**). Densitometric analysis is normalized for the corresponding vinculin content and expressed as fold change related to C SCR (**B**,**D**). Body weight (**E**), GSN (**F**) and TA (**G**) weight correlation to BNIP3 protein expression. Data are means ± SD of 6 mice in the control group and 10 mice in the C26 group. Statistical significance: ** *p* < 0.01 vs. C SCR.

In line with previous observations from our group [14], BNIP3 protein levels are increased in the TA muscle of C26-bearing mice compared to control ones (Figure 1A,B). Inversely, the ratio between free LC3B (LC3B-I and phosphatidylethanolamine-conjugated LC3B (LC3B-II) was not affected, suggesting there was autophagosome accumulation in muscles of the C26 group (Figure 1C,D). Interestingly, BNIP3 protein levels showed negative correlations with body weight (Figure 1E), GSN muscle mass (Figure 1F) and TA muscle mass (Figure 1G), indicating that BNIP3 accumulation is associated with muscle wasting severity.

### 3.2. Electroporation-Mediated BNIP3 Silencing Does Not Prevent BNIP3 Overexpression in the Skeletal Muscle of Tumor-Bearing Mice

**Figure 2:**
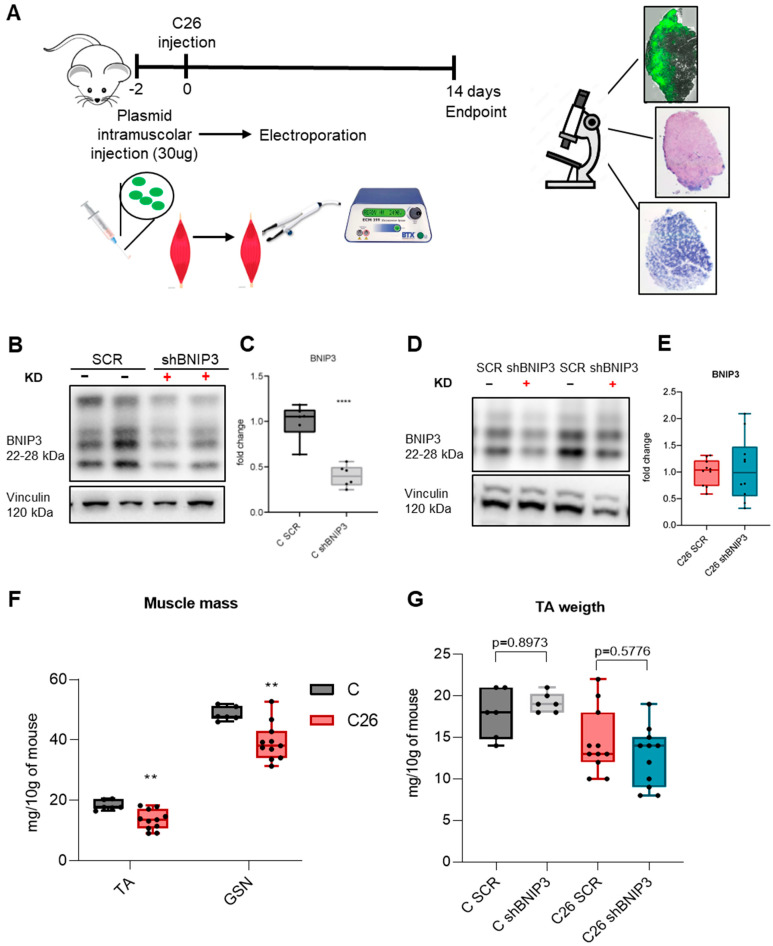
Silencing BNIP3 through electroporation does not prevent its overexpression in the skeletal muscle of tumor-bearing mice. Scheme of experimental protocol and timeline (**A**). Representative immunoblotting showing the expression of BNIP3 in TA muscle (**B**,**D**). Densitometric analysis is normalized for the corresponding vinculin content and expressed as fold of C SCR (**C**) and C26 SCR (**E**), respectively. Differences in tibialis anterior (TA) and gastrocnemius (GSN) muscles (**F**) expressed in milligrams and normalized for 10 g of body weight. TA weight expressed in mg (**G**). Data are means ± SD of 6 mice in the control group and 11 mice in the C26 group. Statistical significance: ** *p* < 0.01, **** *p* < 0.0001 vs. C SCR.3.3. Adenovirus-Mediated BNIP3 Silencing Improves Muscle Atrophy, Preserving Mitochondrial Homeostasis and Oxidative Capacity.

**Figure A1:**
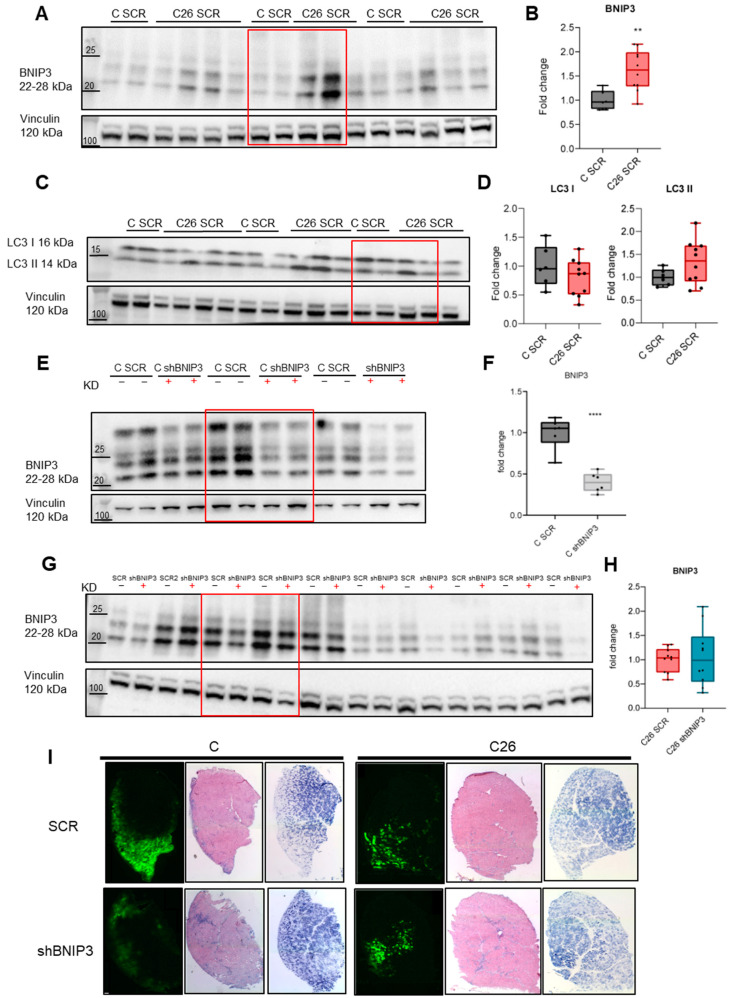
Total representative immunoblotting showing the expression of BNIP3 (**A**,**E**,**G**), LC3 I/II (**C**) in skeletal muscle protein extracts. Densitometric analysis is normalized for the corresponding vinculin content and expressed as fold of C SCR (**B**,**D**,**F**) and C26 SCR (**H**). Representative images at 10× magnification of GFP, H&E and SDH staining performed on tibialis anterior cryostatic section (**I**). Red boxes highlight the representative immunoblotting presented in the main text. Data are means ± SD of 6 mice in the control group and 11 mice in the C26 group. Statistical significance: ** *p* < 0.01, **** *p* < 0.0001 vs. C SCR.

To investigate whether blocking BNIP3-regulated mitophagy could prevent muscle wasting in cancer cachexia, BNIP3 was knocked down in the TA muscle via electroporation of a specific shRNA construct (Figure 2A), a well-established in vivo genetic manipulation [32] previously optimized also in our laboratory for reducing the expression of atrophy-related genes in C26-bearing mice [14]. In order to assess the success of gene delivery, BNIP3 protein expression was evaluated in healthy mice, observing a significant decrease in muscles receiving the specific shRNA compared to the SCR ones (Figure 2B,C and Figure A1E,F). Unfortunately, the same result obtained in controls was not confirmed in C26 TB mice, where no differences were observed at the endpoint (Figure 2D,E and Figure A1G,H). This was potentially due to the strong increase in BNIP3 expression, which may have overcome the silencing capacity of this approach. The marked BNIP3 increase is associated with a severe cachectic condition, as suggested by the significant muscle mass loss (Figure 2F) in C26 TB mice. Moreover, TA weight was reduced (Figure 2G) independently from the scramble or the BNIP3-specific sequence electroporation (32 ± 9 mg versus 29 ± 9 mg, *p* = 0.5776), indicating that the current attempt to block BNIP3 did not protect against muscle loss. H&E and SDH stainings suggest that the gene delivery had no effect on either muscle morphology or oxidative characteristics (Figure A1).

**Figure 3:**
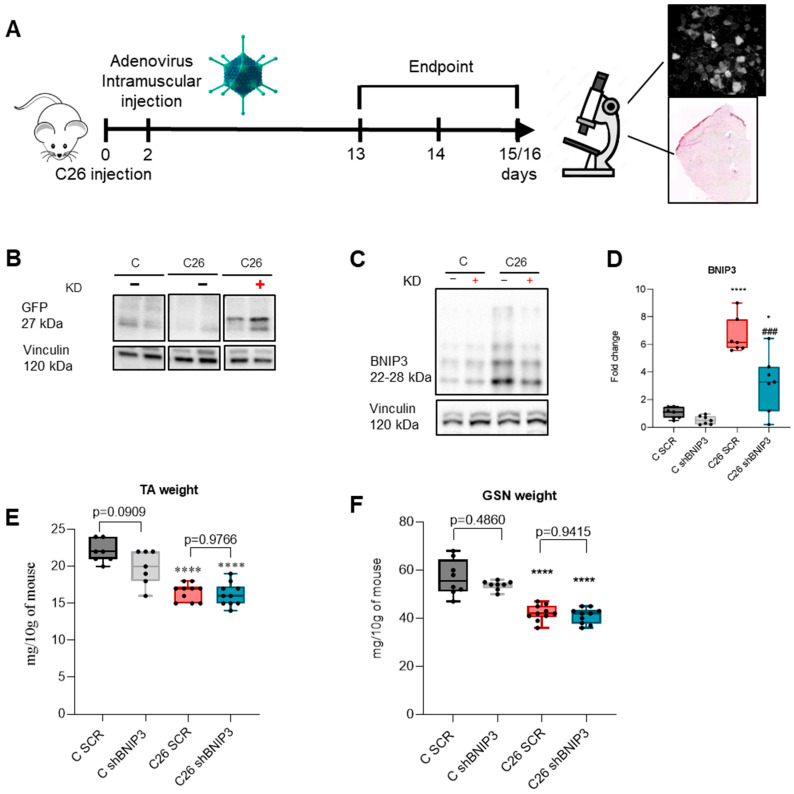
Adenovirus-mediated BNIP3 silencing prevents BNIP3 overexpression in the skeletal muscle of C26 hosts. Scheme of experimental protocol and timeline (**A**). Representative immunoblotting showing the expression of GFP (**B**) and BNIP3 (**C**) in GSN skeletal muscle protein extracts. Densitometric analysis is normalized for the corresponding vinculin content and expressed as fold of C SCR (**D**). Tibialis anterior (TA) and gastrocnemius (GSN) muscles (**E**,**F**), weight expressed in mg and normalized for 10 g of body weight. Data are means ± SD of 6–8 mice in the control group and 9–10 mice in the C26 group. Statistical significance: * *p* < 0.05, **** *p* < 0.0001 vs. C SCR and ### *p* < 0.001 vs. C26 SCR.

**Figure A2:**
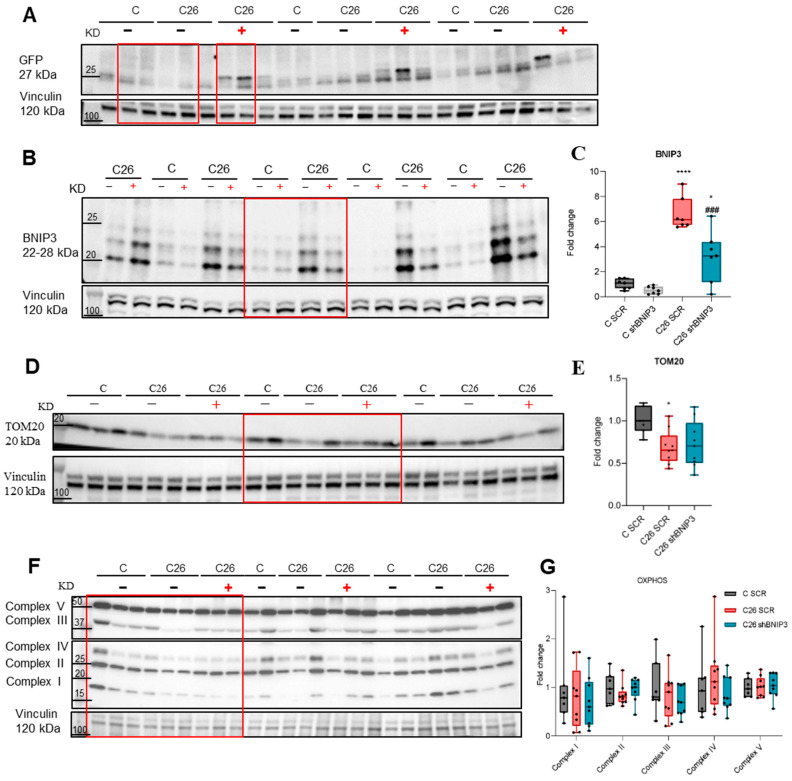
Representative immunoblotting showing the expression of GFP (**A**) and BNIP3 (**B**) in GSN, TOM20 (**D**) and complexes of mitochondrial respiratory chain (**F**) in TA skeletal muscle. Densitometric analysis is normalized for the corresponding vinculin content and expressed as fold change related to C SCR (**C**,**E**,**G**). Red boxes highlight the representative immunoblotting presented in the main text. Data are means ± SD of 6–8 mice in the control group and 9–10 mice in the C26 group. Statistical significance: * *p* < 0.05, **** *p* < 0.0001 vs. C SCR and ### *p*< 0.001 vs. C26 SCR.

Given that BNIP3 silencing via electroporation was not sufficient to counter the marked increase in BNIP3 levels observed in C26 mice, we decided to test another approach of gene delivery using an adenovirus, as previously implemented by Irazoki and colleagues [31] (Figure 3A). To confirm the success of gene delivery and based on the GFP cassette exclusively in the Ad-shBNIP3 vector, GFP protein levels in GSN homogenates were evaluated, confirming an evident 27 KDa GFP band only in the muscles receiving the Ad-shBNIP3 (Figure 3B and Figure A2A). The data shown in Figure 3C,D and Figure A2B,C suggest that the adenoviral delivery was effective in reducing BNIP3 protein expression levels in both the control and C26 groups. Moreover, the decrease was particularly significant in C26 mice, where BNIP3 levels are higher as compared to controls, although not reaching a complete normalization to control values. TA and GSN weight were reduced in C26-bearing mice independently from BNIP3 targeting (Figure 3E). In detail, no changes were observed in the TA mass of either control mice (51 ± 5 mg versus 46 ± 7 mg, *p* = 0.0909) or TB mice (38 ± 4 mg versus 37 ± 4 mg, *p* = 0.9766). Similar results were observed in the GSN muscles (Figure 3F) for control mice (134 ± 24 mg versus 126 ± 12 mg, *p* = 0.4860) and TB mice (98 ± 9 mg versus 95 ± 10 mg, *p* = 0.9415).

**Figure 4:**
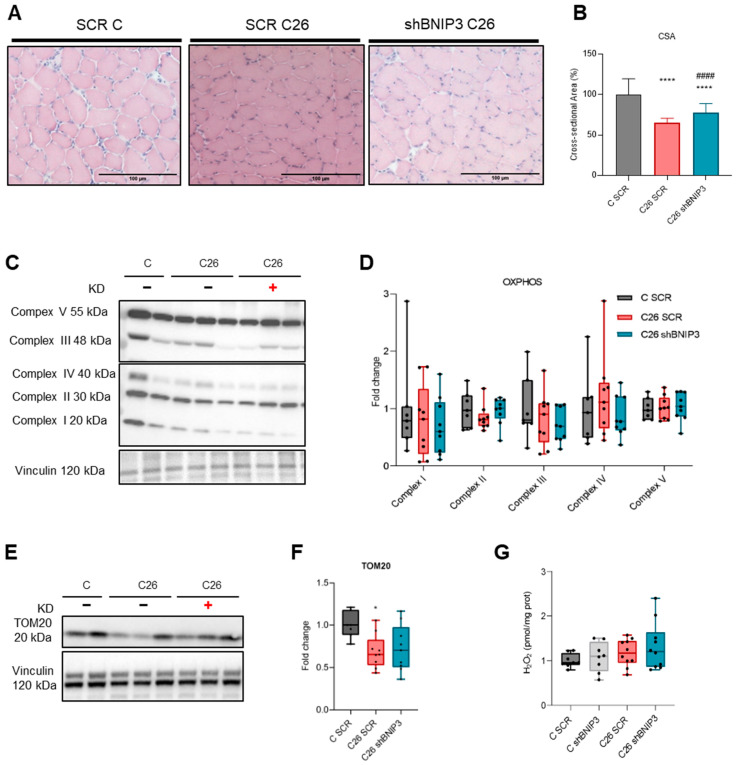
BNIP3 silencing via adenovirus improves muscle atrophy while preserving mitochondrial homeostasis and oxidative capacity. Representative images at 10× magnification of H&E staining performed on tibialis anterior cryostatic sections (**A**). Myofiber cross-sectional area (CSA) of TA muscles expressed as % relative to the C SCR group (**B**). Representative immunoblotting showing the expression of complexes of mitochondrial respiratory chain (**C**) and TOM20 (**E**) in TA skeletal muscle protein extracts. Densitometric analysis is normalized for the corresponding vinculin content and expressed as fold of C SCR (**D**,**F**). Evaluation of H2O2 levels in GSN protein extracts (**G**) expressed as pmol per milligram of protein. Data are means ± SD of 6–8 mice in the control group and 9–10 mice in the C26 group. Statistical significance: * *p* < 0.05, **** *p* < 0.0001 vs. C SCR and #### *p* < 0.0001 vs. C26 SCR.

Fiber cross-sectional area (CSA) was then measured on TA transversal sections and, despite the evidence of a decrease in fiber area in both C26 TB mice groups, which represents a common hallmark of muscle atrophy, a slight improvement in the C26 group receiving the shBNIP3 compared to the control group was observed (Figure 4A,B), indicating a beneficial effect on muscle wasting.

**Figure A3:**
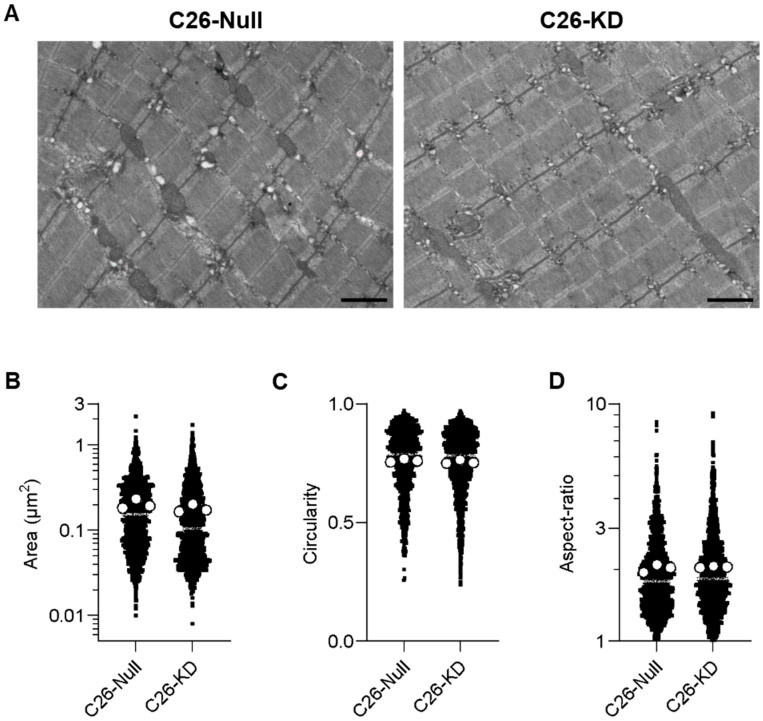
Representative images of subsarcolemmal mitochondria of gastrocnemius muscles obtained with transmission electron microscopy (**A**); quantification of area (**B**), circularity (**C**) and aspect-ratio (**D**) parameters of mitochondria. White circles correspond to mean values/animal (*n* = 3), whereas small grey circles correspond to every individual measurement. Statistical analysis: two-tailed Student’s *t*-test.

Considering the BNIP3 overexpression in the muscle of C26 hosts and the evidence of its role in mitochondrial function, the next question was whether BNIP3 knockdown would impact mitochondrial homeostasis in C26 hosts. For this purpose, the protein expression of mitochondrial respiratory chain complexes (OxPhos) was assessed in TA skeletal muscle and, as shown in Figure 4C,D, no differences were found, suggesting that the mitochondrial oxidative machinery was not affected by acute BNIP3 knockdown. These data were accompanied by a slight increase in TOM20 protein levels in the muscles receiving the Ad-shBNIP3 (Figure 4E,F), suggesting a trend towards increased mitochondrial mass upon BNIP3 downregulation. On the other hand, alterations in mitochondrial turnover are frequently associated with reactive oxygen species accumulation, affecting the redox status and finally leading to oxidative stress. However, H_2_O_2_ levels analyzed in GSN homogenates as a measure of oxidative stress did not significantly differ among the groups, implying that acute BNIP3 downregulation does not impair oxidative balance (Figure 4G). Indeed, a semi-quantitative electron microscopy analysis showed that the mitochondrial morphologies of BNIP3-silenced and SCR-infected skeletal muscles did not differ significantly (Figure A3A–D).

## 4. Discussion

Previous studies using experimental models of cancer cachexia have shown that altered energy homeostasis and muscle proteolysis induction in the skeletal muscle of tumor hosts contribute to muscle mass depletion. Structural and functional alterations of the mitochondrial pool were reported in both humans and mice undergoing cachexia, leading to reduced oxidative phosphorylation capability and ROS accumulation accompanied by increased mitochondrial disposal by mitophagy [5,14,29,30,33,34,35]. However, blocking the autophagic flux in cancer hosts, for example with colchicine [5], a microtubule-destabilizing agent that interacts with tubulin, causes muscle lysosomal engulfment and autophagosome accumulation, ultimately resulting in animals’ death. Such evidence reinforces the idea that a complete blockade of autophagy should not be pursued in cancer hosts already experiencing systemic energy failure. Mitophagy can be either beneficial or detrimental to the process of muscle wasting, and carefully modulating only excessive mitophagy, without prolonging the inhibition of other forms of autophagy for too long, could instead have a protective effect by preserving the muscle mitochondrial pool, potentially improving muscle mass and function. Indeed, blocking BNIP3-mediated mitophagy chronically using a specific HIF1α inhibitor leads to increased mitochondrial damage and metabolic collapse [36]. However, Macleod and colleagues demonstrated that a specific mitophagy defect does not result in the same consequences as seen in complete autophagy inhibition [23], although these observations were limited to a cancer-specific environment. Indeed, in BNIP3-deficient tumors, p62 accumulation was not observed, as mitophagy is reduced but not entirely suppressed [37]. This may be due to compensatory mechanisms involving other mitophagy regulators, such as NIX or Parkin [37]. Zhang and colleagues recently highlighted BNIP3’s significant role as the mitophagy receptor in PGAM5-mediated muscle wasting, showing that disrupting the PGAM5-BNIP3 complex does not negatively impact skeletal muscle health [38]. Muscle mass loss in whole-body BNIP3 knockout mice upon colorectal MC38 or pancreatic ductal Pan02 carcinoma was prevented, supporting the potential of targeting BNIP3 for fighting cancer cachexia, although the chronic and ubiquitous BNIP3 deficit does not allow for the dissection of the specific contribution of muscle mitophagy to the resulting phenotype [38].

Given the lack of conclusive data, this study aimed to clarify the role of BNIP3-mediated mitophagy in muscle wasting associated with cancer, testing whether muscle-specific BNIP3 silencing may counteract muscle atrophy in tumor-bearing mice. The marked increase of BNIP3 levels in the muscle of C26 TB mice supports and reinforces previous findings, suggesting mitophagy overactivation and providing a good model for mechanistic loss of function studies. In the first experimental approach adopted, the electroporation of a shBNIP3-GFP plasmid vector into the skeletal muscle successfully knocked down BNIP3 protein levels in healthy control mice but was ineffective in C26 hosts, where the cancer-induced BNIP3 overexpression was not abrogated with this gene transfer technique. It is worth mentioning that using the same silencing strategy, i.e., the same plasmid backbone, electroporation protocol and tumor model, although targeting different genes such as muscle-specific ubiquitin ligases, was effective in counteracting muscle fiber atrophy [39], supporting the idea that the experimental system is potentially adequate; however, the induction of BNIP3 overcame the current silencing capacity. Distinct results were obtained with the use of an adenoviral vector targeting BNIP3, which successfully achieved BNIP3 knockdown even in the skeletal muscle of C26 hosts. BNIP3 silencing was unable to counteract macroscopic C26-induced muscle depletion, as shown by the lack of differences in muscle mass, partially ascribable to the incomplete suppression of BNIP3 protein accumulation, suggesting that the knockdown occurred only in a portion of the muscle fibers. Interestingly, fiber cross-sectional area was assessed, and despite both C26 TB mice groups exhibiting a reduction in fiber area, which represents a common hallmark of muscle atrophy in C26 hosts, there was a slight improvement in the C26 Ad-shBNIP3 group compared to the control. This suggests a beneficial effect of BNIP3 knockdown in ameliorating muscle atrophy, although the partial effect as compared to the greater impact described in the abovementioned study [38] requires some specifications. In the current study, BNIP3 knockdown was performed acutely, locally and using a model producing severe cachexia, as opposed to chronic and whole-body BNIP3 loss in mice bearing tumors that produce mild wasting. Fortunately, both approaches resulted in a beneficial effect, providing a rationale for further studies aimed at developing a pharmacological approach.

It is worth reminding that Irazoki and colleagues in 2022 demonstrated that BNIP3 knockdown in myotubes leads to mitophagy inhibition and lysosomal alterations, finally resulting in the disruption of mitochondrial dynamics [31], raising concerns on the potentially detrimental effects of chronic mitophagy inhibition. The association between the impairment of mitophagy and the accumulation of damaged and dysfunctional mitochondria is well established, resulting in increased ROS production and oxidative stress [15,36]. However, the results obtained in the present study suggest that short-term, partial BNIP3 knockdown in C26 hosts does not negatively impact mitochondrial homeostasis or oxidative capacity, as indicated by SDH staining and the unchanged levels of mitochondrial respiratory complexes. This finding is supported by the expression levels of the mitochondrial mass marker TOM20. Although TOM20 was significantly reduced in the skeletal muscle of C26 TB mice, BNIP3 silencing not only prevented further reduction but also slightly preserved its levels. In aged mice, mitochondrial dysfunction is associated with BNIP3 accumulation and increased ROS production [31]. In such a condition, BNIP3 downregulation further exacerbated mitochondrial damage and contributed to muscle atrophy, suggesting that in chronic aging sarcopenia, increased BNIP3 represents an adaptive response aimed at removing damaged mitochondria. Conversely, in the current acute experimental setting, H_2_O_2_ levels in the skeletal muscle of C26 hosts were not impacted by BNIP3 knockdown, supporting the idea that BNIP3 silencing may mitigate excessive mitophagy in skeletal muscle without adversely affecting the oxidative balance, which remains preserved. Consistently, oxidative stress has been reported to take part in the pathogenesis of cancer-induced muscle wasting, although not playing a critical role [40]. Because the study was conducted at two separate research institutes, a limitation of this study could be differences in experimental conditions, such as the gender and the age of the mice, specified in the protocol’s methods, producing consistent degrees of muscle atrophy within the same timeframe. On the other hand, there is no established consensus on a standardized procedure for using the C26 model. A further limitation of this study is represented by the lack of mitochondrial function assessments, such as ATP production or O_2_ consumption, which require further investigation in the future.

Finally, the different roles of BNIP3 in cancer and in host tissues require further investigation, as its function appears to be tissue-specific and varies across different tumors and molecular contexts [28]. In line with the controversial BNIP3 role, we would like to highlight the need for any future systemic translational approaches (e.g., pharmacological) aimed at targeting BNIP3 in experimental cancer models or patients to carefully consider the potential effects and outcomes on all tissues, whether normal or tumoral.

## 5. Conclusions

Mitochondrial dysfunction and excessive mitophagy are found in various disease states characterized by muscle wasting, including cancer cachexia. Here we propose a model in which silencing BNIP3 improves muscle atrophy while preserving mitochondrial homeostasis and the endogenous control of redox balance. In conclusion, although deeper analyses and alternative approaches targeting mitophagy are warranted, the current dataset suggests that selectively and partially blocking BNIP3 overexpression to prevent excessive mitochondrial disposal represents a promising therapeutic goal for counteracting muscle wasting in cancer patients.

## Data Availability

All data are included in this paper and the Supplementary Information. Raw data underlying figures are available upon request to the corresponding author. No new omics data, requiring repository deposit, were generated.

## Supplementary Materials

The following supporting information can be downloaded at: https://www.mdpi.com/article/10.3390/cancers16244133/s1, Figure S1: WB densitometry.
