# Supplementary material for: BNIP3 Downregulation Ameliorates Muscle Atrophy in Cancer Cachexia
Source: Cancers (Basel). 2024 Dec 11;16(24):4133. doi: 10.3390/cancers16244133 (PMC11674865; doi:10.3390/cancers16244133)
Supplement: Supplementary file 1 [file cancers-16-04133-s001.zip › cancers-3331590-supplementary.pdf]

FIG 1A-B	BNIP3	Vinculin
C SCR	3022308	1901824
C SCR	3232404	2114320
C26 SCR	2856204	1618648
C26 SCR	6106446	1642588
C26 SCR	6004620	1463855
C26 SCR	4299354	1707017
C SCR	4550616	2057054
C SCR	3497040	1915276
C26 SCR	8848710	2430879
C26 SCR	7955190	2415318
C26 SCR	4239504	1839086
C SCR	3583710	1923047
C SCR	4296384	1718607
C26 SCR	6334380	1532293
C26 SCR	5177520	2053919
C26 SCR	7127712	2429226

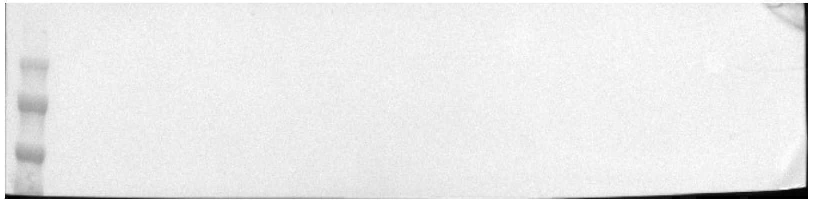

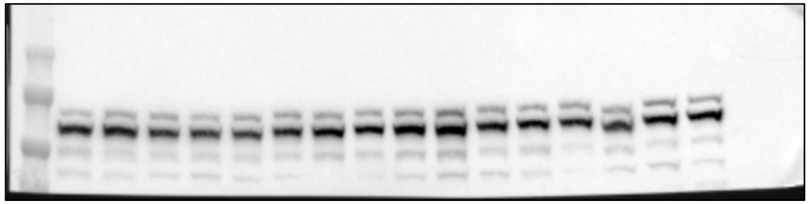

Vinculin

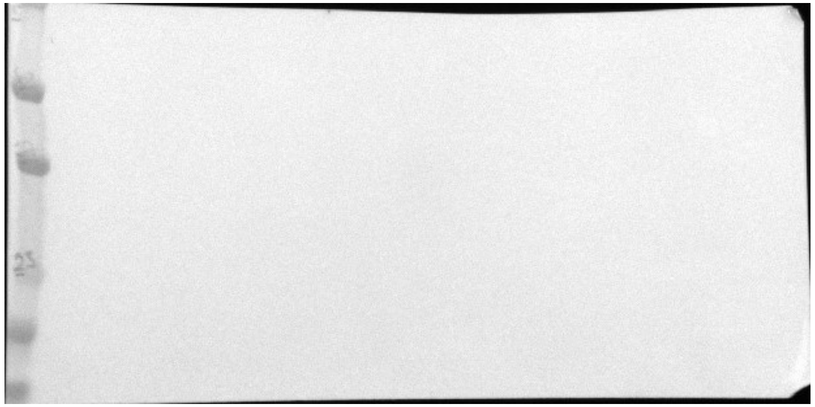

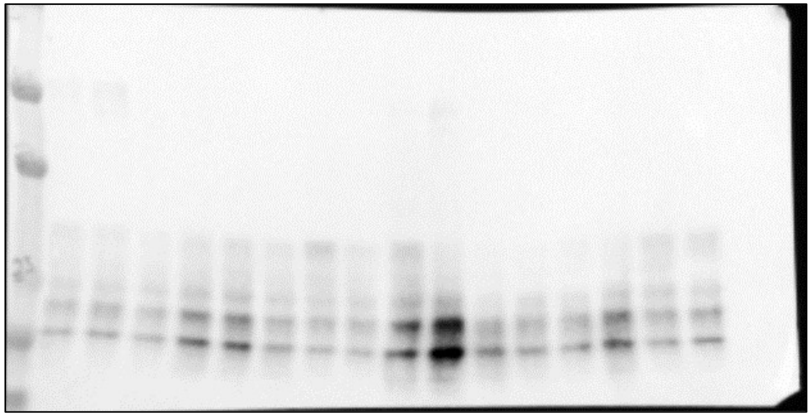

BNIP3

Fig 1C-D	LC3 I 16 kDa	Vinculin
C SCR	1344850	1445717
C SCR	1744912	1820026
C26 SCR	1301898	1014663
C26 SCR	605358	1170039
C26 SCR	1319552	1530204
C26 SCR	1377428	1267331
C SCR	1044758	1423773
C SCR	778440	1435486
C26 SCR	634218	1938131
C26 SCR	2217800	2136628
C26 SCR	1612260	1890395
C SCR	2544022	1684501
C SCR	1868802	1497119
C26 SCR	1679704	1802372
C26 SCR	931216	1958073
C26 SCR	1028846	1835626

	LC3 II 14 kDa	Vinculin
C SCR	1043458	1445717
C SCR	1227928	1820026
C26 SCR	842270	1014663
C26 SCR	2205658	1170039
C26 SCR	1614418	1530204
C26 SCR	1629212	1267331
C SCR	1280214	1423773
C SCR	1560182	1435486
C26 SCR	2683902	1938131
C26 SCR	3148184	2136628
C26 SCR	1609660	1890395
C SCR	1646840	1684501
C SCR	1223300	1497119
C26 SCR	2619708	1802372
C26 SCR	1285492	1958073
C26 SCR	1112540	1835626

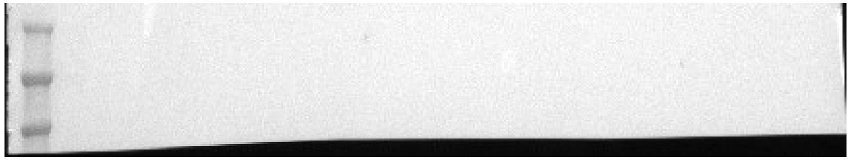

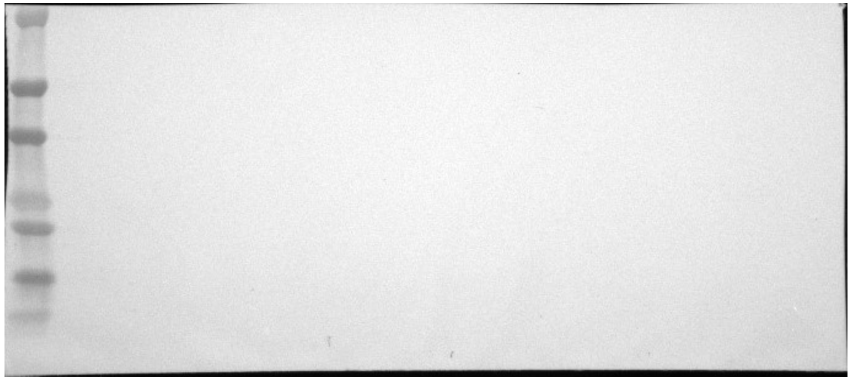

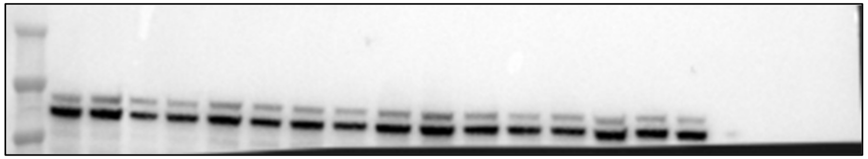

Vinculin

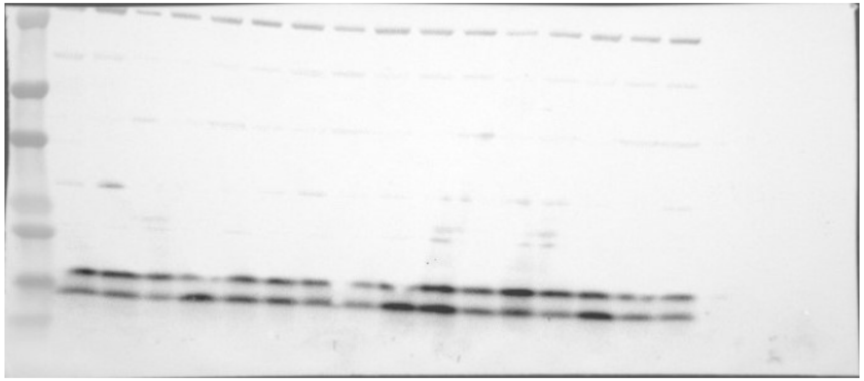

LC3

Fig 2B-C

BNIP3	22-28 kDa	Vinculin
C SCR	4151112	2103718
C SCR	4809664	2289673
C shBNIP3	3334686	3923505
C shBNIP3	3381703	4126008
C SCR	6099600	5380979
C SCR	6426629	3439503
C shBNIP3	3132288	3148752
C shBNIP3	3264876	5498696
C SCR	4215312	2476404
C SCR	3889889	2076844
C shBNIP3	1427150	3210480
C shBNIP3	1631340	2939378

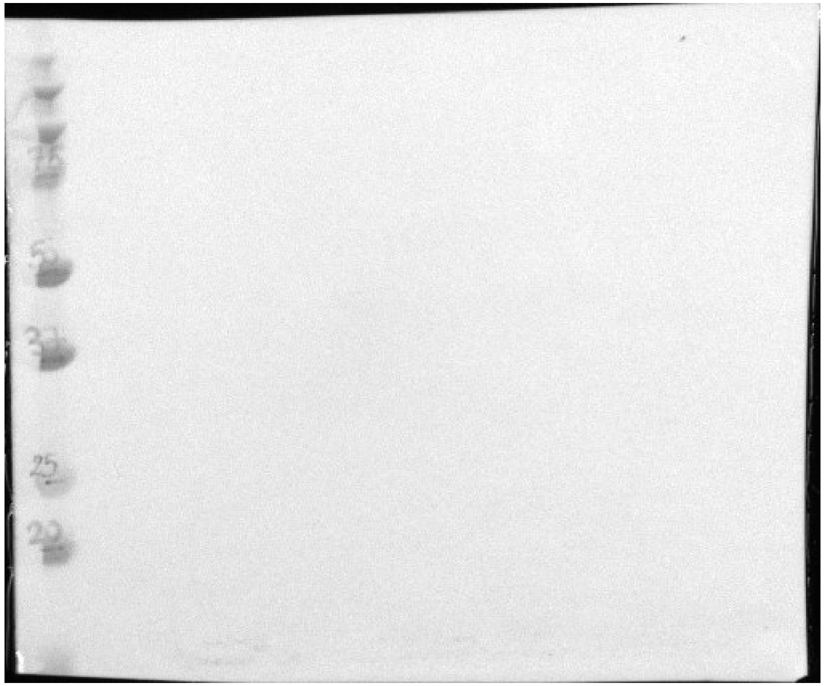

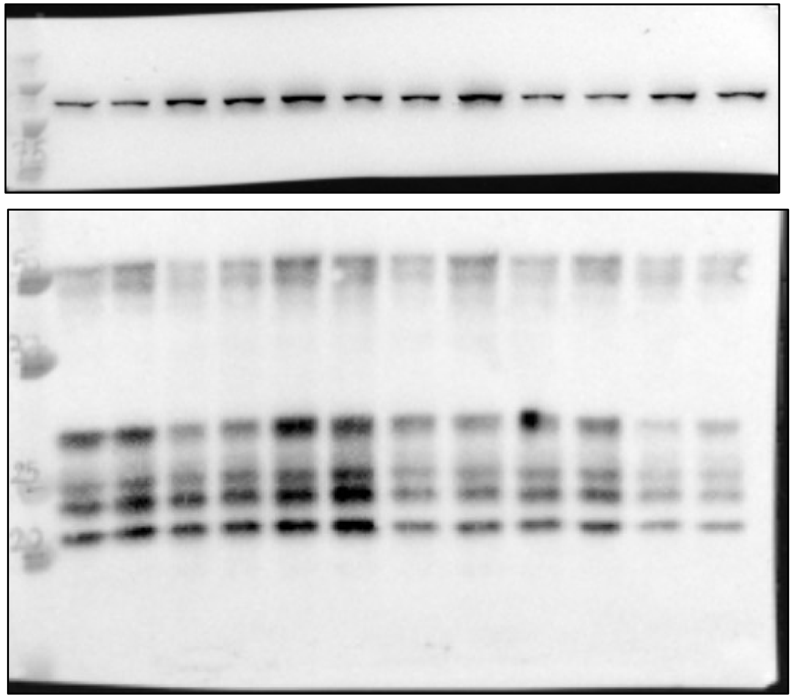

Vinculin

BNIP3

Fig 2D-E

BNIP3	22-28 kDa	Vinculin
C26 SCR	1303296	1188552
C26 shBNIP3	1121124	1435896
C26 SCR	2661612	1397556
C26 shBNIP3	3104280	1389264
C26 SCR	2657604	1318980
C26 shBNIP3	2039496	1397712
C26 SCR	3331896	1358160
C26 shBNIP3	3100572	792708
C26 SCR	3589548	1486680
C26 shBNIP3	3421956	962328
C26 SCR	1304232	970728
C26 shBNIP3	1246080	866136
C26 SCR	1921440	1020708
C26 shBNIP3	1067484	974352
C26 SCR	1376736	990648
C26 shBNIP3	2147172	931584
C26 SCR	2043336	914784
C26 shBNIP3	2677248	1072872
C26 SCR	1971360	998664
C26 shBNIP3	674988	1135428

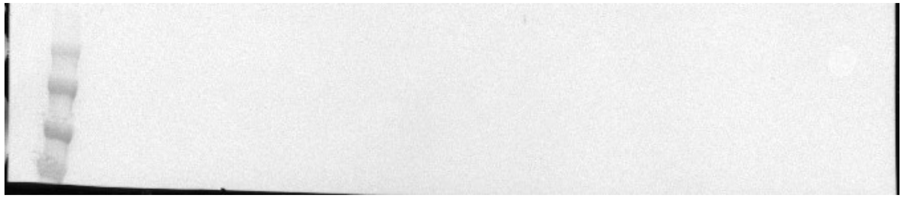

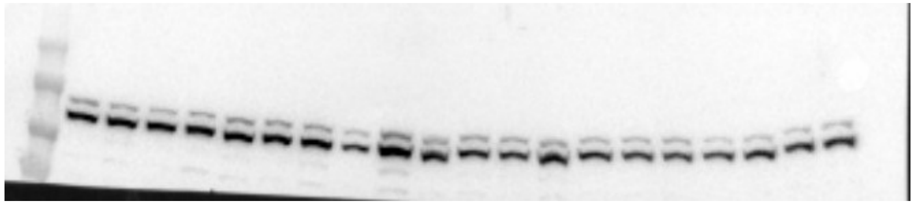

Vinculin

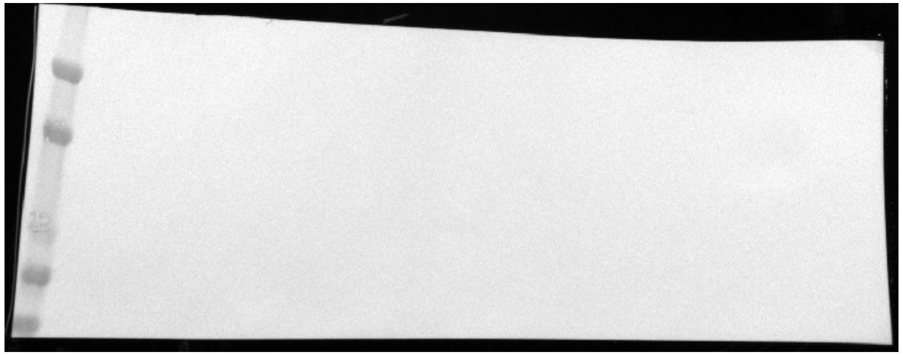

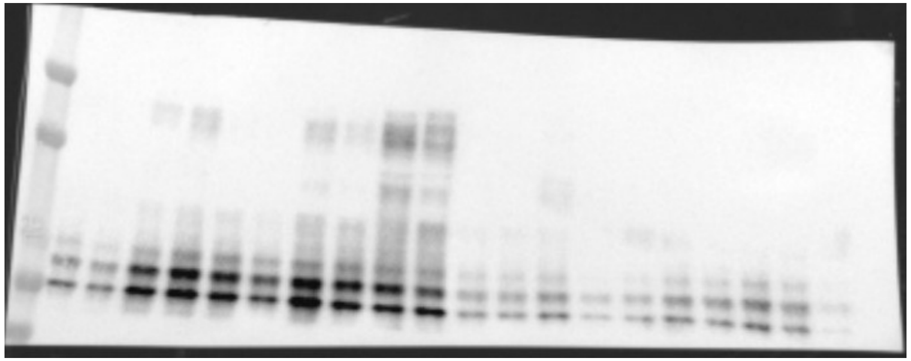

BNIP3

Fig 3C-D	BNIP3	Vinculin
C26 SCR	1550882	1308996
C26 shBNIP3	61671,32	2133820
C SCR	336192,6	1902500
C shBNIP3	205771,8	1973349
C26 SCR	1700325	1826100
C26 shBNIP3	485584,6	2774536
C SCR	226793,7	2216122
C shBNIP3	55239,61	2182606
C26 SCR	1726814	1979523
C26 shBNIP3	1011480	2047480
C SCR	198882	1929220
C shBNIP3	117771,4	1671369
C26 SCR	1860769	2206500
C26 shBNIP3	1129961	1968620
C SCR	428927,9	1910986
C shBNIP3	46530,34	1556289
C26 SCR	1832958	2110437
C26 shBNIP3	1050568	2205380
C SCR	499818,5	2255072
C shBNIP3	258012,2	1824722
C26 SCR	2324783	2469000
C26 shBNIP3	1240671	1871394
C SCR	174028,1	2497360
C shBNIP3	71519,46	1674698
C26 SCR	3529747	2586936
C26 shBNIP3	2295621	2357187
C SCR	388401,4	2373840
C shBNIP3	291927,7	2417028

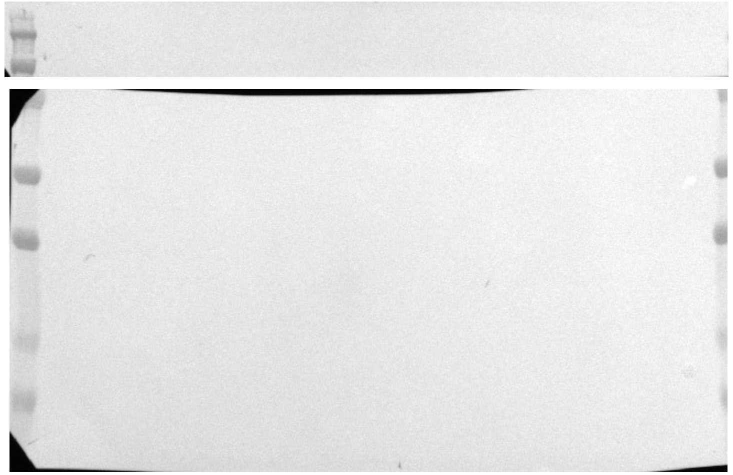

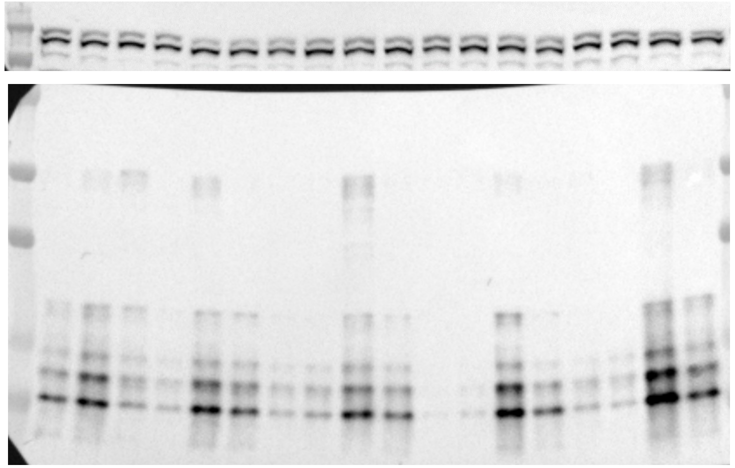

Vinculin

BNIP3

I	OXPHOS	Vinculin
C SCR	3632412	396024
C SCR	1368756	412884
C SCR	1039656	366828
C26 scr	932520	356412
C26 scr	399108	371304
C26 scr	501360	349332
C26 shBNII	566928	339996
C26 shBNII	318264	381216
C26 shBNII	202284	311640
C SCR	446844	286200
C SCR	795624	374592
C26 scr	74544	289320
C26 scr	58848	253236
C26 scr	959328	320040
C26 shBNII	119244	344820
C26 shBNII	1095312	329268
C26 shBNII	1608456	423300
C SCR	320952	382128
C SCR	1407420	560604
C26 scr	1399416	446928
C26 scr	3134064	567780
C26 scr	2620800	478104
C26 shBNII	1918848	832596
C26 shBNII	735900	383436
C26 shBNII	1867956	365052

II	OXPHOS	Vinculin
C SCR	4017696	396024
C SCR	2997720	412884
C SCR	2930784	366828
C26 scr	2492280	356412
C26 scr	1884132	371304
C26 scr	2203992	349332
C26 shBNII	3229368	339996
C26 shBNII	3372132	381216
C26 shBNII	2959296	311640
C SCR	3501996	286200
C SCR	3501156	374592
C26 scr	2336676	289320
C26 scr	1677984	253236
C26 scr	3560100	320040
C26 shBNII	2566428	344820
C26 shBNII	3234768	329268
C26 shBNII	3466728	423300
C SCR	1971936	382128
C SCR	3038412	560604
C26 scr	2558292	446928
C26 scr	3280488	567780
C26 scr	3225264	478104
C26 shBNII	3020556	832596
C26 shBNII	2382804	383436
C26 shBNII	3018156	365052

III	OXPHOS	Vinculin
C SCR	2427624	396024
C SCR	950460	412884
C SCR	1026492	366828
C26 scr	1190064	356412
C26 scr	234984	371304
C26 scr	267948	349332
C26 shBNII	725820	339996
C26 shBNII	607692	381216
C26 shBNII	683124	311640
C SCR	706836	286200
C SCR	1732764	374592
C26 scr	529824	289320
C26 scr	444624	253236
C26 scr	1642440	320040
C26 shBNII	494004	344820
C26 shBNII	1056648	329268
C26 shBNII	1395252	423300
C SCR	364992	382128
C SCR	1282104	560604
C26 scr	1243800	446928
C26 scr	1826796	567780
C26 scr	1633728	478104
C26 shBNII	1221708	832596
C26 shBNII	345240	383436
C26 shBNII	1207164	365052

IV	OXPHOS	Vinculin
C SCR	1495560	396024
C SCR	501096	412884
C SCR	647184	366828
C26 scr	1119420	356412
C26 scr	520668	371304
C26 scr	613104	349332
C26 BNIP3	826524	339996
C26 BNIP3	772836	381216
C26 BNIP3	767232	311640
C SCR	1054488	286200
C SCR	2656188	374592
C26 scr	1247160	289320
C26 scr	1221408	253236
C26 scr	2894100	320040
C26 BNIP3	733740	344820
C26 BNIP3	1503108	329268
C26 BNIP3	1601100	423300
C SCR	593232	382128
C SCR	1638504	560604
C26 scr	1056972	446928
C26 scr	2378484	567780
C26 scr	1675368	478104
C26 BNIP3	1578396	832596
C26 BNIP3	439512	383436
C26 BNIP3	1403064	365052

V	OXPHOS	Vinculin
C SCR	4671924	396024
C SCR	4055676	412884
C SCR	3965820	366828
C26 scr	4022640	356412
C26 scr	3310152	371304
C26 scr	3053436	349332
C26 shBNII	4353180	339996
C26 shBNII	3748632	381216
C26 shBNII	4504404	311640
C SCR	4120308	286200
C SCR	4958772	374592
C26 scr	3644076	289320
C26 scr	3525804	253236
C26 scr	4881492	320040
C26 shBNII	3250596	344820
C26 shBNII	4737132	329268
C26 shBNII	4571196	423300
C SCR	3424332	382128
C SCR	5029680	560604
C26 scr	4985748	446928
C26 scr	5289696	567780
C26 scr	5440836	478104
C26 shBNII	5225640	832596
C26 shBNII	4431108	383436
C26 shBNII	5286312	365052

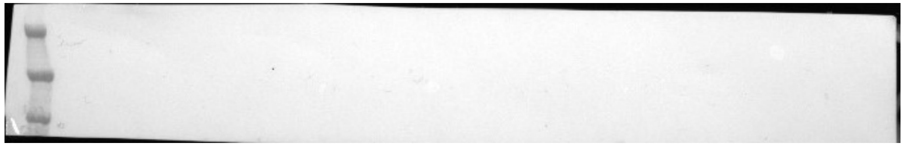
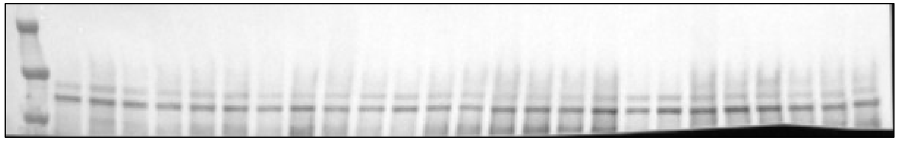

Vinculin

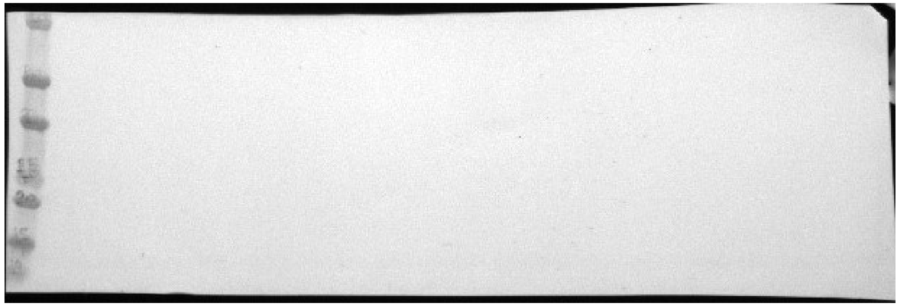
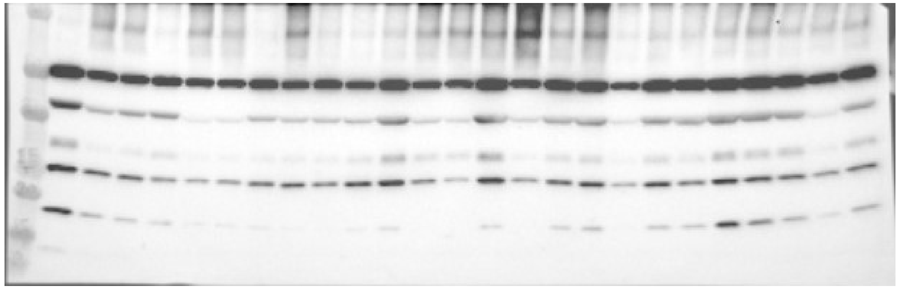

OXPHOS

Fig 4E-F	TOM20	Vinculin
C SCR	2085746	1730911
C SCR	1866306	1671930
C SCR	2145481	1697852
C26 scr	1673061	1840098
C26 scr	941070	1530243
C26 scr	905073	1644864
C26 shBNIP3	1350583	1910649
C26 shBNIP3	1273896	1931969
C26 shBNIP3	777725	1705821
C SCR	1932567	1974778
C SCR	2681874	1802450
C26 scr	1236300	1498744
C26 scr	1006928	1408264
C26 scr	2344160	1758575
C26 shBNIP3	1596036	1448720
C26 shBNIP3	2031302	1488786
C26 shBNIP3	2479919	1686243
C SCR	1732224	1368796
C SCR	2528643	1654588
C26 scr	1000948	1226186
C26 scr	1760343	1491542
C26 scr	1580293	1787214
C26 shBNIP3	1440946	1620307
C26 shBNIP3	830661	1357343
C26 shBNIP3	1729039	1775761

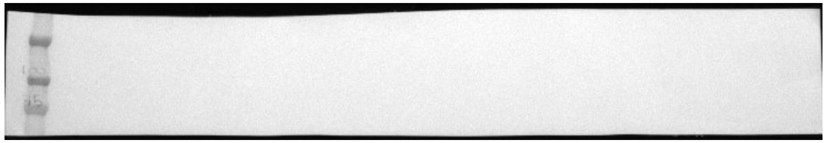

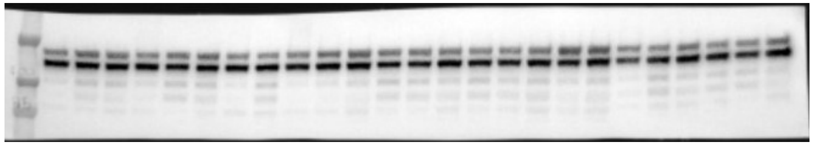

Vinculin

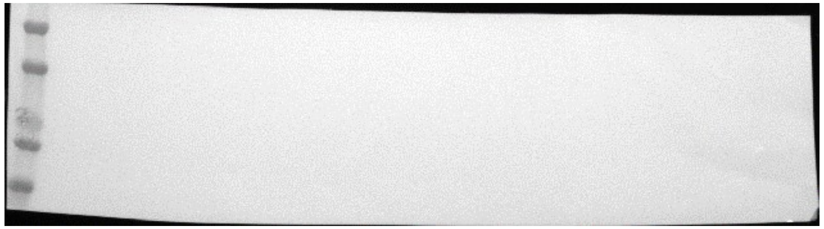

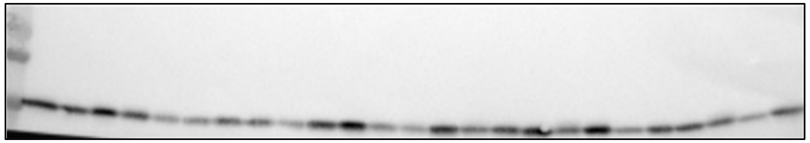

TOM20
